# Zeolite application and irrigation during ripening reduced berry sunburn damage and yield loss in cv. Sangiovese (*Vitis vinifera* L.)

**DOI:** 10.3389/fpls.2024.1427366

**Published:** 2024-07-26

**Authors:** Gianluca Allegro, Gabriele Valentini, Daniela Sangiorgio, Chiara Pastore, Ilaria Filippetti

**Affiliations:** Department of Agricultural and Food Sciences, University of Bologna, Bologna, Italy

**Keywords:** adaptation strategies, anthocyanins, berry composition, climate change, cluster exposure, irrigation

## Abstract

Climate change poses significant challenges to the grapevine cultivation for wine production. In the last years, the occurrence of extreme weather events such as intense heat waves and prolonged period of drought increased sunburn damage with negative consequences to yield and berry composition. Short-term adaptation strategies are urgently needed to mitigate these effects. In this light, our study aimed to evaluate the efficacy of zeolite application on the canopy and irrigation during ripening on sunburn damage, yield, and berry composition in cv. Sangiovese (*Vitis vinifera* L.). Over 3 years (2021–2023), canopy management and irrigation treatments were arranged in a strip-plot design. Canopy management treatments included leaf removal on the cluster zone, with and without zeolite application, and no defoliated control; irrigation treatments comprised irrigation from berry softening and no irrigation. Both zeolite application and irrigation reduced sunburn damage, thus mitigating yield loss. Regarding berry composition, zeolite application did not alter the main parameters whereas irrigation led to reductions in sugar and anthocyanin concentrations. These findings suggest that zeolite application and irrigation during ripening represent promising and cost-effective solutions for grape growers facing climate change-induced challenges. However, further studies are necessary to optimize timing of irrigation to avoid detrimental effects on anthocyanin accumulation.

## 1 Introduction

Like other perennial crops, grapevine needs to face serious challenges due to the intensification of extreme weather events caused by climate change, including heat waves, drought, and irregularities in both the frequency and intensity of rainfall (Droulia and Charalampopoulos, 2022) occurring in many wine-growing areas worldwide. Temperatures above 35°C cause an acceleration of sugar accumulation and a simultaneous reduction in grape acidity (Coombe, 1987), coupled with a modification of secondary metabolites, aroma, and coloration of grapes (Pastore et al., 2017a). In this perspective, viticulture might suffer from extremely high light intensities, high temperature, and UV radiation in summer which promote, in blackberry varieties, both the occurrence of the decoupling of technological and phenolic maturity of berries (Allegro et al., 2020) and the appearance of berry sunburn (Rustioni et al., 2014). Sunburn symptoms can start with brown/necrotic spots on berries epidermis that might cause partial/completed shriveling of grapes and/or lead to complete desiccation (necrosis) of berries (Gambetta et al., 2021). Both ripening decoupling and sunburn are worsened by the fact that higher temperatures advance grapevine phenology (Parker et al., 2011) and thus grapes ripen earlier in the season, under warmer temperatures (Molitor and Junk, 2019). Long-term climate change adaptation strategies might include a shift in the suitable viticulture area and cultivar replacement (Fraga et al., 2012). However, this kind of strategies requires high investments and the relocation of entire agricultural sectors. Therefore, studies on short-term measures aimed at changing viticultural management practices should be prioritized among the scientific community.

Identified by Cronstedt in 1756, zeolites are tectosilicates and encompass 54 different mineral species characterized as hydrated aluminosilicates of alkaline and alkaline earth elements (Passaglia and Sheppard, 2001). Their crystal chemistry imparts unique physical and chemical properties, including high and selective cation exchange capacity, reversible dehydration, selective molecular absorption, and catalytic behavior (Armbruster and Gunter, 2001). Thanks to these characteristics, natural zeolites find numerous applications in agriculture, as highlighted by Eroglu et al. (2017). In particular, rocks containing more than 50% of zeolites can be classified as “zeolitite” specifying the main zeolite constituent (Galli and Passaglia, 2011). Moreover, with the classification of zeolites as “non-toxic” by the International Agency for Research on Cancer (International Agency for Research on Cancer, 1997) and the Food and Drug Administration’s (FDA) classification as “safe” for human consumption (Rotondi et al., 2022), their use in various sectors, including agriculture, is further supported.

While the use of zeolite in soil is widespread (Perez-Caballero et al., 2008; Doni et al., 2024), its application for foliar treatments is less common. However, in addition to serving as a sustainable tool against pests and pathogens (De Smedt et al., 2015; Calzarano et al., 2019), it has been demonstrated that when sprayed on grapevine canopy, zeolites can reduce berry temperature (Valentini et al., 2021). Therefore, their use for limiting sunburn appearance should be evaluated. Although post-veraison irrigation is not applied in the Mediterranean Basin, in California it was proved that the cautious application of water during berry ripening might prevent yield loss (Mendez et al., 2011). Indeed, it has been observed that high temperatures and prolonged water stress promote the occurrence of berry necrosis and berry shrivel (Bonada et al., 2013).

In this work, we aimed at evaluating the effect of the application of zeolites and of irrigation, over 3 years, on sunburn damage, yield parameters, and berry composition of the black berry cv. Sangiovese, the most cultivated Italian grapevine.

## 2 Materials and methods

### 2.1 Plant material, experimental design, and treatment application

The study took place in a 9-year-old experimental vineyard of the University of Bologna, located in Cadriano, Italy (44°32′N, 11°22′E). Vines were *Vitis vinifera* L. cv. Sangiovese, clone 12T grafted onto SO4 rootstock (*Vitis riparia × Vitis berlandieri*), spaced 1 m within the row and 2.8 m between rows, which were oriented northeast to southwest. The vineyard was established in a loamy soil (40% sand, 36% loam, 24% clay) with an average content of organic matter (1.40%) and low active limestone (0.50%). Vines were trained to a vertical shoot positioned (VSP) spur-pruned cordon. Winter pruning left six two-bud spurs, and during spring, 12 shoots per vine were left. Therefore, at the phenological phase BBCH 79—majority of berries touching (Lorenz et al., 1995)—the number of clusters was uniformed leaving 16 clusters per vine. The disease control program over the season was according to the Integrated Pest Management (IPM) Guidelines of the Emilia-Romagna Region.

The experiment was conducted over 3 years (2021–2023) on a total of 90 vines, and treatments were laid out in a strip-plot design with three blocks and five replicates for each block (N = 15). The two main factors were canopy management and irrigation. Canopy management treatments were (a) removal of main and lateral leaves from the eight basal nodes of each shoot (LR) at the beginning of veraison (DOY 209 in 2021 and 2022, DOY 214 in 2023); (b) no leaf removal control (C); and (c) leaf removal performed as in LR treatment coupled with zeolite sprayings (LR+ZEO). Italian chabazite-rich zeolite (Zeover, Verdi, Reggio Emilia, Italy) was mixed with water (3 kg h L^−1^) and applied to both sides of the whole canopy using a knapsack sprayer (model M3, Cifarelli, Pavia, Italy) immediately after leaf removal. We decided to perform such a drastic leaf removal at the beginning of veraison, when the berries are particularly susceptible to sunburn, to evaluate the effects of zeolite sprays and irrigation during ripening as strategies to mitigate the occurrence of sunburn symptoms. Irrigation treatments were (d) irrigation from the beginning of berry softening—prior to the development of the variety-specific color—until harvest, with water supplied every week to maintain vines well-watered (WW), and (e) no irrigation applied (NI). The amount of water provided was assessed following the irrigation guidance according to the Decision Support System (DSS) Manna Irrigation Intelligence. In essence, Manna Irrigation utilizes a Kc-t (crop coefficient versus time) graph to identify crop stages and gauge the progression of Kc for the current season. Subsequently, utilizing satellite measurements of NDVI, the actual Kc is computed to fix the estimated Kc. Through the application of this methodology, Manna Irrigation calculates the water balance and formulates irrigation recommendations (Koliopanos et al., 2023).

### 2.2 Climate data

A weather station (Davis Instruments, Hayward, CA, USA) installed in the vineyard provided data of air temperature and precipitation from 1 April to 31 October of each year.

### 2.3 Assessment of stem water potential and berry temperature

After veraison, midday stem water potential (Ψ_stem_) was gauged on nine mature leaves for each canopy management × irrigation treatments combination. This was accomplished on three separate days (DOY 236 in 2021, DOY 223 in 2022, DOY 236 in 2023) performed in correspondence to intense heat waves, using a Pump-Up pressure chamber (PMS Instruments, Albany, OR, USA) after wrapping the leaves in plastic film and aluminum foil 2 h prior to the measurements. Berry temperature was measured with an infrared thermometer (model Raynger ST, Raytek, Santa Cruz, CA, USA) in 2022 and 2023, in the same days of stem water potential assessment. Measurements were taken between 1 PM to 2 PM on three berries per tagged vine, placed in the southeast side of the canopy, which at that time of the day was exposed to the sunlight.

### 2.4 Sunburn damage assessment

The incidence and severity of berry sunburn necrosis and berry shrivel were assessed by visual inspection: the proportion of damaged clusters (incidence) and the percentages of berries showing the symptoms (severity) were recorded at harvest (DOY 263 in 2021 and 2022, DOY 269 in 2023) on all the tagged vines. Sunburn necrosis was recorded when berries become necrotic (Gambetta et al., 2021), whereas berry shrivel was recorded when berries were partially dehydrated and appeared like a deflated soccer ball (Bondada and Keller, 2012).

### 2.5 Berry sampling, yield components, composition parameters at harvest, and pruning wood

At harvest, two batches of samples were collected from each tagged vine, one of 30 berries to assess total soluble solid concentration (TSS), pH, and titratable acidity (TA) and one of 20 berries to assess anthocyanin concentration. *Botrytis cinerea* severity on clusters was visually estimated after clusters were removed from the vine. Then, the yield of each vine was weighed and the number of clusters counted. At the end of each season, in February 2022, 2023, 2024, all the tagged plants were pruned by hand and the wood was weighed.

TSS was measured with a temperature-compensating Maselli R50 refractometer (Maselli Misure, Parma, Italy), whereas pH and TA were measured with a Crison Titrator (Crison Instruments, Barcelona, Spain). Anthocyanin extraction, as well as analysis, was performed, following the method described in Mattivi et al. (2006). This involves using 100% methanol as the extraction solvent. For each sample, the skins of 20 berries were removed, weighed, and placed in 100 mL of methanol, in the dark for 24 h. Extracted anthocyanins were analyzed using a Waters 1525 HPLC (Waters, Milford, MA) equipped with a diode array detector and a Phenomenex reversed-phase column with pre-column (Phenomenex, Castel Maggiore, Italy). Anthocyanins were quantified at 520 nm using an external calibration curve with malvidin-3-glucoside chloride as the standard (Sigma-Aldrich, St. Louis, MO, USA) as described by Mattivi et al. (2006).

### 2.6 Statistical analysis

All data were subjected to ANOVA over the years using the mixed procedure available in SAS v9.0 (SAS Institute, Cary, NC, USA). Canopy management treatments and irrigation levels and years were compared using the Tukey’s honestly significant difference with mean separation at P ≤ 0.05.

## 3 Results

**Figure 1:**
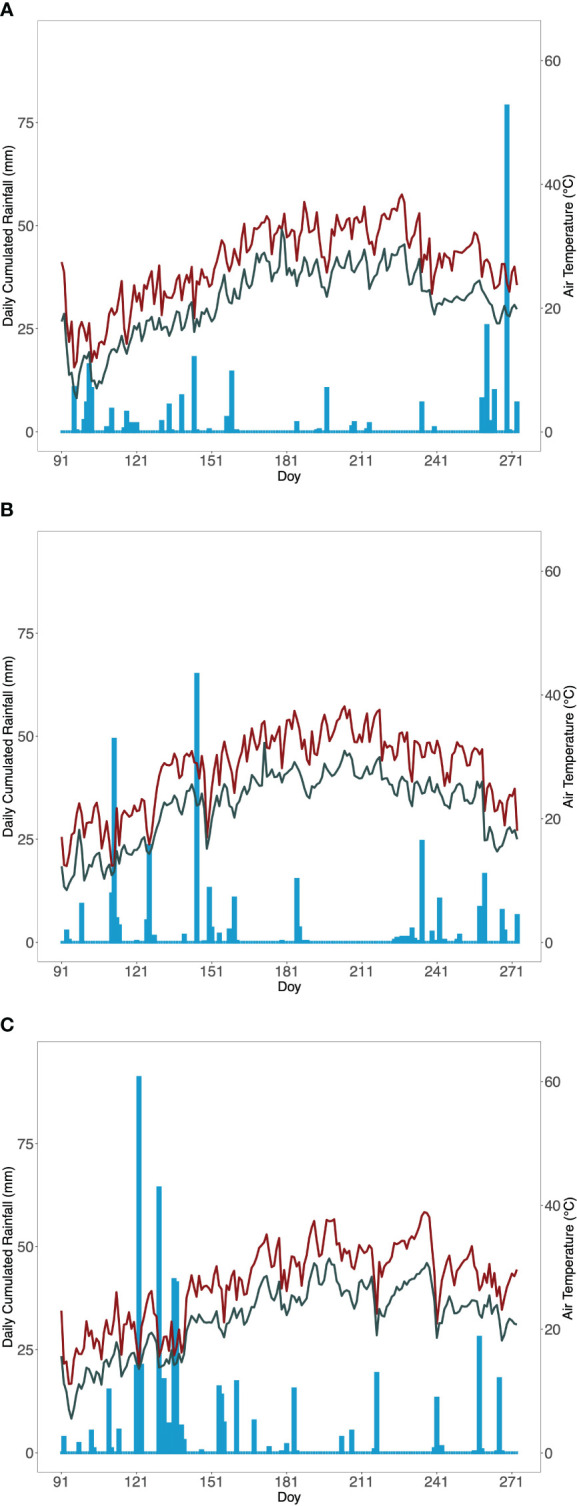
Weather evolution for **(A)** 2021, **(B)** 2022, and **(C)** 2023 seasons (from April to October). Bars show cumulated daily rainfall; red and green lines represent daily maximum and mean temperature, respectively.

Precipitation from April to September was much higher in 2023 (528 mm) compared with 2021 (283 mm) and 2022 (243 mm), mainly due to the heavy rainfall (more than 300 mm fell within 20 days) that occurred in May 2023 (**Figure 1**). In the 3 years of the experiment, few rainfall events occurred in the period from veraison to harvest and the most relevant (>20 mm) were recorded on DOY 261, 235, and 217 in 2021, 2022, and 2023, respectively. The summer periods were characterized by the occurrence of intense and prolonged heat waves that in the period from veraison to harvest increased air temperatures to values higher than 35°C in 9, 5, and 6 days in 2021, 2022, and 2023 (**Figure 1**), respectively. Mean air temperatures of the whole period (from veraison to harvest) were quite similar between the years and ranged between 24.4°C to 24.8°C.

Vine water status (Ψ_stem_) did not change between canopy management treatments, whereas it was differently affected by the irrigation in the 3 years; in fact, only in 2021, Ψ_stem_ of NI vines was significantly lower than that of WW (**Table 1**).

**Table 1:** Effects of canopy management and irrigation treatments on midday stem water potential of Sangiovese leaves (N = 9).

	Ψ_stem_ DOY 236 year 2021 (bar)	Ψstem DOY 223 year 2022 (bar)	Ψstem DOY 236 year 2023 (bar)
Canopy management treatment			
**LR**	−5.90	−3.09	−4.25
**LR+ZEO**	−5.63	−3.18	−4.00
**C**	−5.54	−3.33	−5.08
***Significance***	ns	ns	ns
Irrigation treatment			
**NI**	−7.70 b	−3.61	−4.67
**WW**	−3.68 a	−2.79	−4.22
***Significance***	*	ns	ns
**CM × I**	ns	ns	ns

*P < 0.05; ns, not significant. Different letters within a column indicate a significant difference after Tukey’s honestly significant difference test. Ψ_stem_, midday stem water potential; LR, leaf removal at the beginning of veraison; LR+ZEO, leaf removal + zeolite sprays at the beginning of veraison; C, no leaf removal control; WW, irrigation from the beginning of berry softening; NI, no irrigation; CM, canopy management treatments; I, irrigation treatments; CM × I, canopy management treatments × irrigation treatments interaction.

Berry temperature measured in 2022 and 2023 showed very similar results: as expected, LR increased berry temperature compared with C whereas LR+ZEO values were slightly lower than LR, although not significantly (**Table 2**). Interestingly, irrigation during ripening (WW) reduced berry temperatures of 1.6°C and 1°C in 2022 and 2023, respectively.

**Table 2:** Effects of canopy management and irrigation treatments on the temperature of Sangiovese berries (N = 15).

	Berry temperature on DOY 223 year 2022 (°C)	Berry temperature on DOY 236 year 2023 (°C)
Canopy management treatment		
**LR**	41.4 a	43.5 a
**LR+ZEO**	40.4 a	42.7 a
**C**	35.9 b	35.9 b
***Significance***	***	***
Irrigation treatment		
**NI**	40.0 a	41.6 a
**WW**	38.4 b	40.6 b
***Significance***	*	*
**CM × I**	ns	ns

*P < 0.05; ***, P < 0001; ns. not significant. Different letters within a column indicate a significant difference after Tukey’s honestly significant difference test. LR, leaf removal at the beginning of veraison; LR+ZEO, leaf removal + zeolite sprays at the beginning of veraison; C, no leaf removal control; WW, irrigation from the beginning of berry softening; NI, no irrigation; CM, canopy management treatments; I, irrigation treatments; CM × I, canopy management treatments × irrigation treatments interaction.

The assessment of berry sunburn damage conducted at harvest showed that, as expected, leaf removal caused the occurrence of both berry necrosis and shrivel whereas no symptom was recorded on C clusters. Zeolite treatments limited sunburn damages lowering the severity of berry necrosis in 2021 and 2022 and berry shrivel severity only in 2022 (**Tables 3**, **4**), whereas no effect on the severity of both symptoms was noted in 2023 (**Table 5**). The incidence of berry necrosis was reduced by zeolite treatments only in 2023, whereas that of berry shrivel was reduced by zeolite treatments in all years, even if different levels of this parameter were recorded between years. Additionally, irrigated vines showed lower necrosis severity compared with those not irrigated in the 3 years of the trial.

**Table 3:** Effects of canopy management treatments and irrigation treatments on berry sunburn damages on Sangiovese clusters at harvest of 2021 (N = 15).

	Berry necrosis incidence (%)	Berry necrosis severity (%)	Berry shrivel incidence (%)	Berry shrivel severity (%)
Canopy management treatment				
**LR**	51.6 a	15.9 a	33.7 a	3.8 a
**LR+ZEO**	46.0 ab	8.2 b	8.0 b	1.7 ab
**C**	0.0 b	0.0 c	0.0 b	0.0 b
***Significance***	**	**	**	*
Irrigation treatment				
**NI**	33.4	10.0 a	15.2	2.0
**WW**	31.6	6.0 b	12.5	1.6
***Significance***	ns	*	ns	ns
**CM × I**	ns	ns	ns	ns

*P < 0.05; **P < 0.01; ns, not significant. Different letters within a column indicate a significant difference after Tukey’s honestly significant difference test. LR, leaf removal at the beginning of veraison; LR+ZEO, leaf removal + zeolite sprays at the beginning of veraison; C, no leaf removal control; WW, irrigation from the beginning of berry softening; NI, no irrigation; CM, canopy management treatments; I, irrigation treatments.

**Table 4:** Effects of canopy management treatments, irrigation treatments on berry sunburn damages on Sangiovese clusters at harvest of 2022 (N = 15).

	Berry necrosis incidence (%)	Berry necrosis severity (%)	Berry shrivel incidence (%)	Berry shrivel severity (%)
Canopy management treatment				
**LR**	66.6 a	11.7 a	38.1 a	2.5 a
**LR+ZEO**	63.2 a	7.8 b	9.5 b	0.4 b
**C**	0 b	0 c	0 b	0 b
***Significance***	***	**	**	**
Irrigation treatment				
**NI**	43.8	7.2 a	15.6	0.9
**WW**	42.7	5.7 b	16.2	1.1
***Significance***	ns	*	ns	ns
**CM × I**	ns	ns	ns	ns

*P < 0.05; **P < 0.01; ***P <0001; ns, not significant. Different letters within a column indicate a significant difference after Tukey’s honestly significant difference test. LR, leaf removal at the beginning of veraison; LR+ZEO, leaf removal + zeolite sprays at the beginning of veraison; C, no leaf removal control; WW, irrigation from the beginning of berry softening; NI, no irrigation; CM, canopy management treatments; I, irrigation treatments.

**Table 5:** Effects of canopy management treatments, irrigation treatments on berry sunburn damages on Sangiovese clusters at harvest of 2023 (N = 15).

	Berry necrosis incidence (%)	Berry necrosis severity (%)	Berry shrivel incidence (%)	Berry shrivel severity (%)
Canopy management treatment				
**LR**	57.4 a	13.4 a	54.6 a	4.4 a
**LR+ZEO**	44.8 b	9.3 a	41.2 b	2.9 a
**C**	0 c	0 b	0 c	0 b
***Significance***	**	**	*	**
Irrigation treatment				
**NI**	36.9	9,7 a	33.9	2.7
**WW**	31.3	5,5 b	30.0	2.1
***Significance***	ns	*	ns	ns
**CM × I**	ns	ns	ns	ns

*P < 0.05; **P < 0.01; ns, not significant. Different letters within a column indicate a significant difference after Tukey’s honestly significant difference test. LR, leaf removal at the beginning of veraison; LR+ZEO, leaf removal + zeolite sprays at the beginning of veraison; C, no leaf removal control; WW, irrigation from the beginning of berry softening; NI, no irrigation; CM, canopy management treatments; I, irrigation treatments.

Leaf removal, both with and without zeolite application, reduced the infections of *Botrytis cinerea* on clusters, whereas no difference was found between the irrigation treatments (**Table 6**). Moreover, lower *Botrytis cinerea* severity was recorded in 2023 compared with the previous years. Yield components were affected by both canopy management and irrigation treatments. Yield and cluster weight were lowered by LR compared with C of about the same extent, whereas zeolite sprayings limited the decrease of cluster weight and so the loss of yield. Irrigation during ripening (WW) also limited the loss of yield, resulting in higher yield and cluster weight compared with NI. Moreover, both parameters resulted lower in 2023 compared with 2021 and 2022. Berry weight behaved similarly to cluster weight in response to canopy management and irrigation treatments, whereas regarding year, in 2023 it resulted to be higher than in 2021 and reached an intermediate value in 2022 (**Table 6**), showing the interaction canopy management × year (**Supplementary Table S1**).

**Table 6:** Effects of canopy management treatments, irrigation treatments and year on Botrytis cinerea incidence and yield components of Sangiovese vines (N = 15).

	Botrytis cinerea severity (%)	Clusters (n°/vine)	Yield (kg/vine)	Cluster weight (g)	Berry weight (g)
Canopy management treatment					
**LR**	1.1 b	15.7	4.47 b	284 b	2.41 b
**LR+ZEO**	0.6 b	15.9	5.00 ab	313 ab	2.50 ab
**C**	3.5 a	16.4	6.24 a	382 a	2.65 a
***Significance***	*	ns	**	**	**
Irrigation treatment					
**NI**	1.8	15.9	4.93 b	310 b	2.44 b
**WW**	1.6	16.1	5.54 a	343 a	2.60 a
***Significance***	ns	ns	*	*	*
Year					
**2021**	2.0 a	16.2	5.50 a	339 a	2.48 b
**2022**	2.3 a	16.7	5.62 a	337 a	2.51 ab
**2023**	0.9 b	15.1	4.58 b	303 b	2.57 a
***Significance***	*	ns	***	**	**
Interactions					
**CM × I**	ns	ns	ns	ns	ns
**I × Y**	ns	ns	ns	ns	ns
**CM × Y**	ns	ns	ns	ns	**
**CM × Y × I**	ns	ns	ns	ns	ns

*P < 0.05; **P < 0.01; ***P < 0001; ns, not significant. Different letters within a column indicate a significant difference after Tukey’s honestly significant difference test. LR, leaf removal at the beginning of veraison; LR+ZEO, leaf removal + zeolite sprays at the beginning of veraison; C, no leaf removal control; WW, irrigation from the beginning of berry softening; NI, no irrigation; CM, canopy management treatments; I, irrigation treatments; Y, year.

TSS at harvest was not affected by canopy management treatments whereas irrigation during ripening determined a lower sugar concentration in WW grapes compared with NI ones (**Table 7**). Differences were also noted between years; in fact, in 2021, the sugar concentration was more than 1°Brix lower than in 2022 and 2023. Juice pH was unaffected by any of the factors under evaluation, whereas TA was lower in LR than in C and an intermediate level was reached in LR+ZEO. Moreover, TA remained unaffected by the irrigation treatments but showed significant variation across years, notably lower in 2022 compared with 2021.The HPLC analysis showed that the anthocyanin concentration was higher in LR berries compared with C and again LR+ZEO was intermediate. A sharp difference was then noted between the two irrigation treatments, as WW reduced the anthocyanin concentration by approximately 26% compared with NI. This parameter changed over the years, being higher in 2022 compared with 2023. Variations of TSS, TA, and anthocyanin concentration across years determined some interactions between the factors analyzed (**Supplementary Table S1**).

**Table 7:** Effects of canopy management treatments, irrigation treatments and year on the composition at harvest of Sangiovese berries (N = 15).

	Total soluble solids (Brix°)	pH	Titratable acidity (g/L)	Anthocyanins (mg/kg of grape)
Canopy management treatment				
**LR**	22.3	3.41	6.85 b	599 a
**LR+ZEO**	22.1	3.39	7.20 ab	571 ab
**C**	22.6	3.40	7.44 a	529 b
***Significance***	ns	ns	*	*
Irrigation treatment				
**NI**	22.7 a	3.39	7.19	631 a
**WW**	22 b	3.42	7.14	502 b
***Significance***	*	ns	ns	*
Year				
**2021**	21.4 b	3.35	7.88 a	574 ab
**2022**	22.9 a	3.59	6.13 c	602 a
**2023**	22.6 a	3.27	7.48 b	523 b
***Significance***	***	ns	***	***
Interactions				
**CM × I**	ns	ns	*	ns
**I × Y**	*	ns	ns	***
**CM × Y**	*	ns	***	ns
**CM × Y × I**	*	ns	ns	ns

*P < 0.05; ***P < 0001; ns, not significant. Different letters within a column indicate a significant difference after Tukey’s honestly significant difference test. LR, leaf removal at the beginning of veraison; LR+ZEO, leaf removal + zeolite sprays at the beginning of veraison; C, no leaf removal control; WW, irrigation from the beginning of berry softening; NI, no irrigation; CM, canopy management treatments; I, irrigation treatments; Y, year.

Pruning weight measured in winter did not vary among treatments, but in 2023, it resulted to be higher than in the previous 2 years (**Table 8**).

**Table 8:** Effects of canopy management treatments and irrigation treatments and year on the pruning weight of Sangiovese vines (N = 15).

	Pruning weight (kg/vine)
Canopy management treatment	
**LR**	0.95
**LR+ZEO**	0.85
**C**	0.91
***Significance***	ns
Irrigation treatment	
**NI**	0.84
**WW**	0.97
***Significance***	ns
Year	
**2021**	0.86 b
**2022**	0.83 b
**2023**	1.05 a
***Significance***	*
Interactions	
**CM × I**	ns
**I × Y**	ns
**CM × Y**	ns
**CM × Y × I**	ns

*P < 0.05; ns, not significant. Different letters within a column indicate a significant difference after Tukey’s honestly significant difference test. LR, leaf removal at the beginning of veraison; LR+ZEO, leaf removal + zeolite sprays at the beginning of veraison; C, no leaf removal control; WW, irrigation from the beginning of berry softening; NI, no irrigation; CM, canopy management treatments; I, irrigation treatments; Y, year.

## 4 Discussion

This study highlighted the potential of Italian rich-chabazite zeolite application and irrigation during ripening as innovative adaptation measures against berry sunburn damage induced by climate change. Our findings revealed promising outcomes in symptom reduction and yield loss limitation.

Late irrigation affected vine water status only in 2021 as WW vines kept Ψ_stem_ higher than −4 bar whereas Ψ_stem_ of NI was lowered to near −8 bar due to the prolonged drought and the heat waves that occurred in August. Although WW and NI displayed significantly different values in 2021, −8 bar does not indicate a water stress condition in Sangiovese vines (Valentini et al., 2022). In 2022, rainfall on DOY 235 prevented a decrease of NI Ψ_stem_ as observed in the previous year. Similarly, in 2023, the abundant precipitations in May and rainfall on DOY 217 prevented NI Ψ_stem_ from falling. Although in 2022 and 2023 vine water status was not affected by irrigation, the WW vines showed a decrease in berry temperatures measured during the occurrence of intense heat waves, probably due to the potential increase in transpiration of well-hydrated berries. This condition, witnessed by the higher berry weight of WW vines, may have facilitated the dissipation of heat (Gambetta et al., 2021). Considering that elevated berry temperatures, combined with intense light exposure, are significant factors contributing to sunburn damage (Hulands et al., 2014), the reduction of berry necrosis noted in the WW group likely stemmed from the decreased berry temperatures. Even if the measurements of berry temperature were not continuously recorded throughout the season, they were taken during the occurrence of heat waves and represent realistic pictures of what happens to the vines in these challenging conditions. The exposition of the clusters to high solar radiation and to temperatures which exceeded 40°C led to the occurrence of berry necrosis and shrivel symptoms on over half of the evaluated clusters. However, zeolite treatment was able to reduce the severity of the sunburn symptoms in particular in 2021 and 2022. In this work, we recorded berry temperature and observed only a slight decrease. We have to point out that these results derive from berry temperatures recorded only twice with an infrared thermometer and thus may not fully explain the reduction in severity symptoms. Indeed, in a previous study conducted on cv. Sangiovese (Valentini et al., 2021), in which berry temperatures were continuously monitored by thermocouples, zeolite applications reduced maximum berry temperature by approximatively 4°C for varying durations, ranging from 7 to 18 days, depending on the climate conditions of the years. Hence, in our current study, zeolite application may have protected berries from high temperatures in the days following its application in 2021 and 2022, limiting the occurrence of the sunburn damage and reducing its severity at harvest (Petoumenou, 2023). In 2023, no heat wave occurred in the 2 weeks following zeolite application (it was registered from DOY 230 to DOY 240), and probably in this time frame, the treatment may have diminished its efficacy in reducing sunburn damage severity. It is worth noticing that C berries were not affected by any sunburn damage, meaning that the basal leaves protected the clusters from excessive solar radiation. Unfortunately, several grape-growing areas have been recently subjected to multiple summer stress conditions, which lead to early basal leaf fall around the cluster zone, mainly around veraison. In respect to our work, literature shows different effects of leaf removal and irrigation on sunburn damage, as reported by Müller et al. (2023). In their study, Riesling vines were defoliated 10 and 25 days after fruit set and a drought stress treatment was imposed from fruit set to veraison. Differently from our Sangiovese experiment, leaf removal before veraison allowed the berries to acclimate, thereby enhancing their resilience to sunburn damage (Morales-Quintana et al., 2020). On the contrary, removing the leaves in a period of excessive temperatures may cause lethal damages to the berries (Lopes et al., 2019). Pre-veraison water stress applied to Riesling vines induced heat tolerance in the berries (Müller et al., 2023), highlighting that irrigation might exert varying effects on sunburn damage depending on the phenological phase of application and the vine water status.

Enhanced cluster exposition implemented by leaf removal (in our study LR and LR+ZEO treatments) typically reduces the severity of *Botrytis cinerea* by diminishing the humidity in the fruit zone (Zoecklein et al., 1992). Indeed, properly timed leaf removal was studied as a tool for managing *Botrytis* bunch rot in Sauvignon Blanc grapevines (Würz et al., 2020). The comparatively higher incidence of this fungus observed in 2021 and 2022, in contrast to 2023, may be attributed to precipitation events occurring twice in the week preceding harvest during the former years, totaling over 25 mm. Conversely, in 2023, only one rainfall event (18 mm) was recorded 2 days before harvest. Given that the number of clusters per vine was uniformed at the phenological phase “majority of berries touching” (BBCH 79), yield variations among different canopy management and irrigation treatments were attributed to changes in cluster weight. Therefore, reductions in yield measured in vines subjected to leaf removal were directly proportional to the decreases of cluster weight. In turn, cluster weight was influenced by sunburn damage and berry weight whereas the reduction of leaf area itself did not affect yield in LR and LR+ZEO vines as the leaf removal was carried out removing already senescent leaves when the clusters had been fully formed. Sunburn damage differently reduced the weight of exposed clusters (LR and LR+ZEO) because necrotic or partially dehydrated berries had lower weight than berries without sunburn symptoms. However, as reported in **Table 4**, even berries without heat injuries exhibited weight variations among treatments. Specifically, LR berry weight was lower compared with C as a consequence of intense solar radiation and very high temperatures, which likely constrained cell elongation owing to heightened fruit transpiration rates (Bergqvist et al., 2001). Conversely, irrigation during ripening mitigated berry heat damage and facilitated berry growth compared with NI vines, resulting in higher cluster weights. It is worth noticing that both zeolite and irrigation treatments, given the protective action on berries in reducing necrosis symptoms, were able to mitigate the yield loss up to approximately 2 t/ha.

The grape composition at harvest was differently influenced by the treatments as TSS was reduced by late irrigation (WW) whereas TA was lowered by LR. Notably, leaf removal at veraison did not result in decreased sugar accumulation, which was likely due to the minimal impact of the already senescent basal leaves on the source–sink balance (Bledsoe et al., 1988). Given the almost null impact of leaf removal at veraison on TSS (Pastore et al., 2017b), the leaf area was not measured. The lower TSS observed in WW was primarily attributed to a dilution effect, as the increase of berry weight was proportionally higher than the decrease of sugar concentration (+6% vs. −3%) whereas the decrease of titratable acidity observed in LR berries was likely caused by enhanced degradation of malic acid in response to the higher temperatures experienced by those exposed berries (Allegro et al., 2019). Albeit it is well documented that the synthesis of anthocyanins is adversely affected by very high temperatures (Pastore et al., 2017b), quite surprisingly, our findings revealed higher concentrations of these compounds in LR berries compared with C. Two factors might have contributed to these unexpected results: (a) the reduction of LR berry weight may have led to a concentration effect favoring anthocyanin accumulation; (b) since a portion of the berries directly exposed to sunlight were affected by sunburn damage, we sampled healthy and less exposed berries that likely experienced lower temperatures compared with their highly exposed counterpart. Conversely, irrigation during ripening led to a reduction of anthocyanin concentration due to dilution effect as WW berries exhibited higher weights compared with NI berries. Furthermore, starting irrigation just before the onset of anthocyanin accumulation might have slowed down the synthesis of these compounds, which on the contrary benefit from mild water stress during this phenological phase (Ollé et al., 2011).

Pruning weight did not change among treatments, as they were implemented when shoot growth had ceased. However, the intense rainfall recorded in May 2023 may have stimulated vigorous vegetative growth, consequently resulting in higher pruning weights compared with the previous years.

## 5 Conclusions

In this study, intense leaf removal was applied at veraison to induce sunburn damage to mimic field conditions occurring in several viticultural areas under climate changes. In these challenging conditions, irrigation from berry softening mitigated the symptoms of sunburn necrosis and berry shrivel, whereas zeolite treatment resulted effective in the first 2 years of the experiment, when it was applied very close to a heat wave. Our results showcase the potential of both approaches in alleviating heat damage and minimizing yield loss. Zeolite application did not adversely affect berry composition, whereas irrigation led to reductions of sugar and anthocyanin concentration. These findings offer valuable insights for grape growers facing climate change-induced sunburn damage as zeolite application emerges as a practical and cost-effective solution for protecting clusters when weather forecasts predict heat waves. On the other hand, irrigation can help maintain berries well-hydrated, but further research is needed to refine and optimize the timing of these interventions to prevent interference with anthocyanin accumulation.

## Data availability statement

The raw data supporting the conclusions of this article will be made available by the authors, without undue reservation.

## Author contributions

GA: Conceptualization, Investigation, Methodology, Writing – original draft. GV: Data curation, Investigation, Writing – review & editing. DS: Data curation, Formal analysis, Investigation, Visualization, Writing – review & editing. CP: Data curation, Formal analysis, Investigation, Writing – review & editing. IF: Conceptualization, Funding acquisition, Methodology, Supervision, Writing – review & editing.
