# Supplementary material for: Zeolite application and irrigation during ripening reduced berry sunburn damage and yield loss in cv. Sangiovese (Vitis vinifera L.)
Source: Front Plant Sci. 2024 Jul 26;15:1427366. doi: 10.3389/fpls.2024.1427366 (PMC11310163; doi:10.3389/fpls.2024.1427366)
Supplement: Supplementary file 1 [file DataSheet_1.pdf]

Supplementary Material

Table S1. Mean values of berry weight, total soluble solids, titratable acidity, anthocyanin concentration and berry weight for canopy management and irrigation treatments of each year (N=15).

		2021			2022			2023		
		**LR**	**LR+ ZEO**	**C**	**LR**	**LR+ ZEO**	**C**	**LR**	**LR+ ZEO**	**C**
**Berry weight (g)**	**NI**	2.38	2.36	2.72	2.08	2.22	2.43	2.21	2.23	2.58
	**WW**	2.66	2.62	2.63	2.30	2.26	2.47	2.35	2.46	2.66
**Total soluble solids (Brix°)**	**NI**	22.1	21.3	22.7	22.3	22.9	23.9	23.3	22.6	23.1
	**WW**	20.8	20.0	21.7	23.2	23.0	22.3	22.1	22.6	22.2
**Titratable acidity (g/L)**	**NI**	7.44	8.26	7.98	6.16	6.44	5.99	7.45	7.32	7.68
	**WW**	7.21	7.85	8.56	5.44	6.23	6.50	7.43	7.10	7.92
**Anthocyanins (mg/kg of grape)**	**NI**	719	647	622	707	670	640	626	533	513
	**WW**	505	490	460	539	532	524	500	554	416

LR, leaf removal at the beginning of veraison; LR+ZEO leaf removal + zeolite sprays at the beginning of veraison; C, no leaf removal control; WW, irrigation from the beginning of berry softening; NI, no irrigation.
